# Two new species of *Karschia* Walter, 1889 from Xizang, China (Solifugae, Karschiidae)

**DOI:** 10.3897/BDJ.12.e129933

**Published:** 2024-09-12

**Authors:** Wenlong Fan, Chao Zhang, Feng Zhang

**Affiliations:** 1 Hebei Basic Science Center for Biotic Interaction, Hebei University, Baoding, Hebei 071002, China Hebei Basic Science Center for Biotic Interaction, Hebei University Baoding, Hebei 071002 China; 2 Key Laboratory of Zoological Systematics and Application, College of Life Sciences, Hebei University, Baoding, Hebei 071002, China Key Laboratory of Zoological Systematics and Application, College of Life Sciences, Hebei University Baoding, Hebei 071002 China

**Keywords:** Camel-spider, morphology, taxonomy

## Abstract

**Background:**

Solifugae, commonly known as camel spiders, sun spiders or wind scorpions, is a relatively understudied order of predominantly nocturnal, cursorial and predatory arachnids, characterised by their formidable two-segmented chelicerae, voracious appetite and impressive speed. It is one of the meso-diverse arachnid orders, comprising 15 families, 144 genera, over 1200 described species and five monotypic fossil genera. *Karschia* Walter, 1889 is a small genus within the solifugid family Karschiidae, which, until now, had contained 32 species distributed in North Africa, Middle East and Central Asia, with 12 of these described from western regions of China.

**New information:**

Two new species of the genus *Karschia* Walter, 1889 are described from Xizang, China: Karschia (Karschia) shannan **sp. nov.** and Karschia (Karschia) trisetalis **sp. nov.** Detailed morphological descriptions, photographs of the bodies and a distribution map of these two new species are provided.

## Introduction

The camel-spider genus *Karschia* Walter, 1889 (Arachnida, Solifugae) was erected and placed in Galeodidae Sundevall, 1833 by Walter (1889), with the type species *Karschiacornifera* Walter, 1889 from Turkmenistan. Kraepelin (1899) erected the subfamily Karschiinae Kraepelin, 1899 under Solpugidae Leach, 1815 and transferred *Karschia* to this new subfamily. Then, Roewer (1933) elevated Karschiinae to the rank of a family. Harvey (2003) further refined the classification by dividing the genus *Karschia* into two subgenera: Karschia (Karschia) Walter, 1889 and Karschia (Rhinokarschia) Birula, 1935, based on morphological characteristics, specifically the presence or absence of a horn-like crest on the male cheliceral fixed finger. Recent research has confirmed that Karschiidae Kraepelin, 1899, belongs within the suborder Boreosolifugae Kulkarni et al., 2023 and can be monophyletic (Kulkarni et al. 2023, Kulkarni et al. 2024). However, despite this clarification at the family level, the relationships within the genus *Karschia* remain unclear. This indicates that further studies are needed to elucidate the diversity and phylogeny within this genus.

Until the present paper, this genus had contained 32 species distributed in North Africa, Middle East and Central Asia, with 12 of these described from China: Karschia (Rhinokarschia) liui Fan et al., 2024a (♂♀, Gansu), Karschia (Rhinokarschia) rhinoceros Birula, 1922 (♂♀, Xinjiang), Karschia (Karschia) birulae Roewer, 1934 (♂♀, Xinjiang), Karschia (Karschia) dingye Fan et al., 2024b (♂♀, Xizang), Karschia (Karschia) lhasa Fan et al., 2024b (♂♀, Xizang), Karschia (Karschia) namling Fan et al., 2024b (♂♀, Xizang), Karschia (Karschia) nubigena Lawrence, 1954 (♂, Xizang), Karschia (Karschia) shigatse Fan et al., 2024b (♂♀, Xizang), Karschia (Karschia) tarimina Roewer, 1933 (♀, Xinjiang), Karschia (Karschia) tibetana Hirst, 1907 (♂♀, Xizang), Karschia (Karschia) tienschanica Roewer, 1933 (♀, Xinjiang) and Karschia (Karschia) zhui Fan et al., 2024b (♂♀, Xizang) (Lawrence 1954, World Solifugae Catalog 2024, Fan et al. 2024a, Fan et al. 2024b).

In this paper, two further new *Karschia* species are described and illustrated from Xizang, China: Karschia (Karschia) shannan sp. nov. and Karschia (Karschia) trisetalis sp. nov., bringing the total for the genus up to 34 species, with 14 species from China, the majority of which are from Xizang.

## Materials and methods

The specimens were collected by hand during the day from under stones and were preserved in 75% and 95% ethyl alcohol, respectively, to meet the requirements of both morphological and molecular biological research. Photographs were taken using a Leica M205A stereomicroscope, equipped with a DFC550 CCD camera, or an Olympus BX53 microscope, equipped with a Kuy Nice CCD camera and were imported into Helicon Focus Ver. 7.0 for stacking. Plates and photographs were edited and retouched using Adobe Photoshop 2022. Drawings were made using the Inkscape software (Ver. 1.0.2.0). All measurements are in milimetres (mm). Pedipalp and leg measurements are shown as: total length (femur, tibia, metatarsus, tarsus). All specimens are deposited in the Museum of Hebei University (**MHBU**), Baoding, China.

Descriptions follow the format of Birula (1935) and Gromov (2004), with some modifications. The terminology used for identifying teeth and other structures in the chelicerae follows Bird et al. (2015). The term ‘ctenidia’ stands for long, single-tipped (non-bifid) and flexible setiform structures present on some opisthosomal sternites.

**Abbreviations as follows: A/CP**, the sum of the lengths of pedipalp, leg I and leg IV divided by the sum of the lengths of chelicera and propeltidium, indicating the length of appendages in relation to body size. Long-legged species have larger A/CP ratios. **CL/CH**, chelicera length/height, large CL/CH ratios suggest a narrow cheliceral morphology, while a more robust morphology is represented by a smaller ratio; **CL**, chelicera length; **CH**, chelicera height; ***fcp*** (modified *pvd*), flagellar complex plumose setae; ***fcs***, flagellar complex subspiniform to spiniform setae; **FD**, fixed finger, distal teeth/tooth; **FM**, fixed finger, medial teeth/tooth; **FP**, fixed finger, proximal teeth/tooth; **FSD**, fixed finger, subdistal teeth/tooth; **FSM**, fixed finger, submedial teeth/tooth; **FST**, fixed finger, subterminal teeth/tooth; ***pdp***, prodorsal proximal setae; **PF**, profondal tooth; **PFM**, profondal medial teeth/tooth; **PFP**, profondal proximal teeth/tooth; **PFSP**, profondal subproximal teeth/tooth; **PH**, propeltidium height; **PL**, propeltidium length; ***pvd***, proventral distal setae; ***pvsd***, proventral subdistal setae; **MM**, movable finger, medial teeth/tooth; **MP**, movable finger, proximal teeth/tooth; **MSM**, movable finger, submedial teeth/tooth; **MSP**, movable finger, subproximal teeth/tooth; **MST**, movable finger, subterminal teeth/tooth; **RF**, retrofondal teeth/tooth; **RFA**, retrofondal apical teeth/tooth; **RFM**, retrofondal medial teeth/tooth; **RFP**, retrofondal proximal teeth/tooth; **RFSP**, retrofondal subproximal teeth/tooth; ***rlf***, retrolateral finger setae; ***sme***, socket margin elevation.

## Taxon treatments

### Karschia (Karschia) shannan sp. nov.

E82E9A5B-819E-5DAD-B375-C64783BAD1CF

86A99BAB-8D68-4E65-8AEA-EF0237744532

#### Materials

- **Type status:** Holotype. **Occurrence:** recordedBy: Xiangbo Guo; sex: male; lifeStage: adult; occurrenceID: 1C596901-1780-5A92-8615-CB5AC70B46E9; **Taxon:** scientificName: Karschia (Karschia) shannan; **Location:** country: China; stateProvince: Xizang Autonomous Region; county: Gongga; municipality: Shannan; locality: Jiangtang Town; verbatimElevation: 3560 m; verbatimCoordinates: 29.3213°N, 90.6874°E; **Identification:** identifiedBy: Wenlong Fan; **Event:** year: 2023; month: July; day: 6; **Record Level:** language: English; institutionID: the Museum of Hebei University (MHBU); institutionCode: MHBU-Sol-XZ2023070601
- **Type status:** Paratype. **Occurrence:** recordedBy: Xiangbo Guo; sex: 1 male, 1 female; lifeStage: adult; occurrenceID: F48EB559-A918-5B3B-933C-9E7D9F1C91AC; **Taxon:** scientificName: Karschia (Karschia) shannan; **Location:** country: China; stateProvince: Xiang Autonomous Region; county: Gongga; municipality: Shannan; locality: Jiangtang Town; verbatimElevation: 3560 m; verbatimCoordinates: 29.3213°N, 90.6874°E; **Identification:** identifiedBy: Wenlong Fan; **Event:** year: 2023; month: July; day: 6; **Record Level:** language: English; institutionID: the Museum of Hebei University (MHBU); institutionCode: 1 male MHBU-Sol-XZ2023070602 1 female MHBU-Sol-XZ2023070603

#### Description

**Figure 1.**
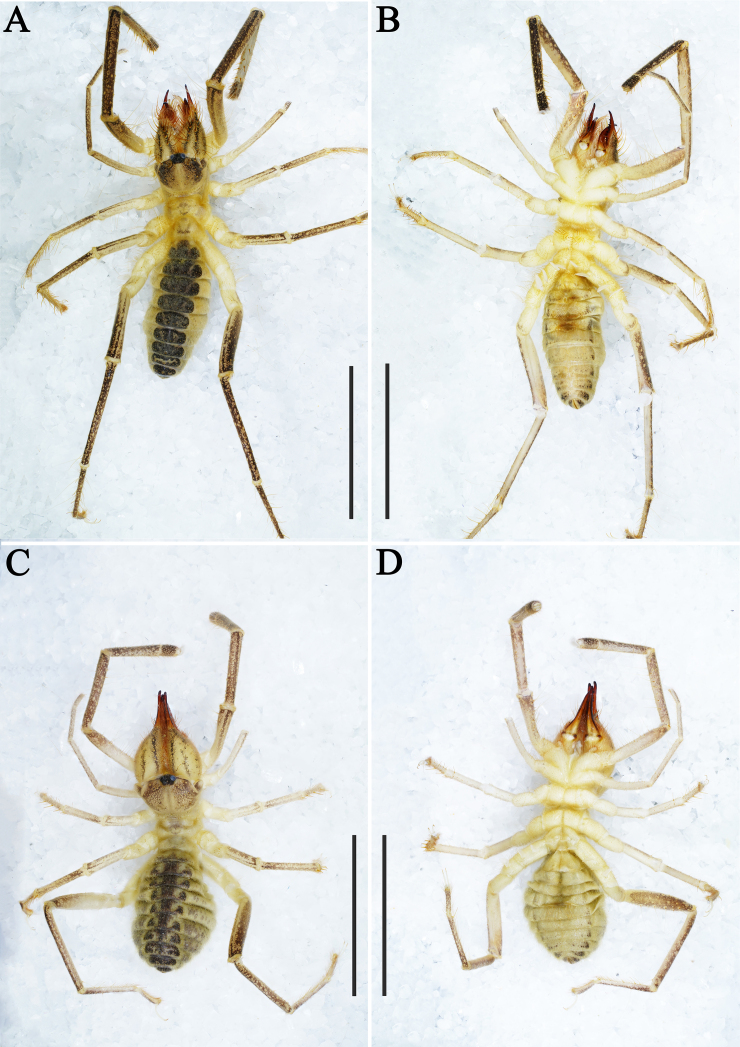
Habitus. Karschia (Karschia) shannan sp. nov., habitus: **A, B** Male holotype; **C, D** Female paratype. Scale bars: 8 mm.

**Figure 2.**
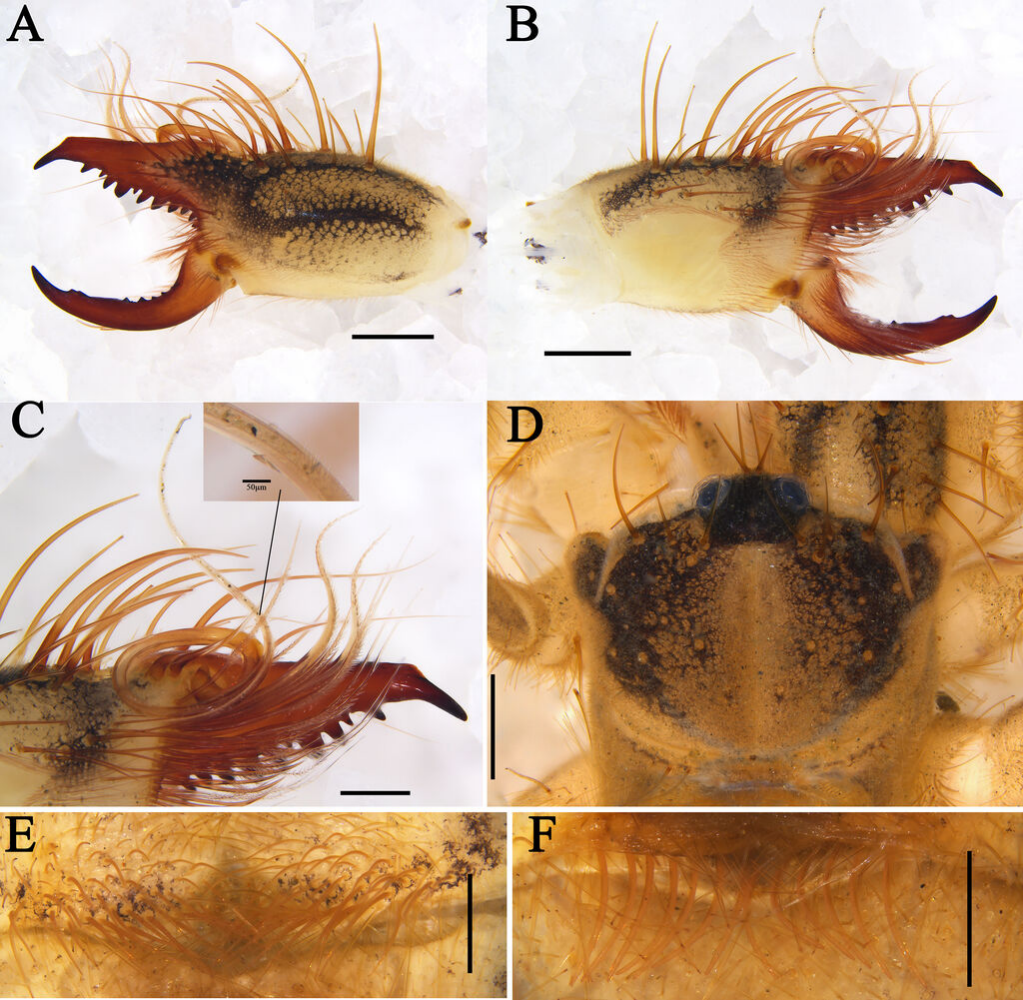
Karschia (Karschia) shannan sp. nov., male holotype: **A** Left chelicerae, retrolateral aspect; **B** Left chelicerae, prolateral aspect; **C** Flagellar complex (inset with magnified view of apophysis), prolateral aspect; **D** Propeltidium, dorsal aspect; **E** Ctenidia on sternite Ⅲ, ventral aspect; **F** Ctenidia on sternite IV, ventral aspect. Scale bars = 1.0 mm (A, B, D); 0.5 mm (C, E, F).

**Figure 3.**
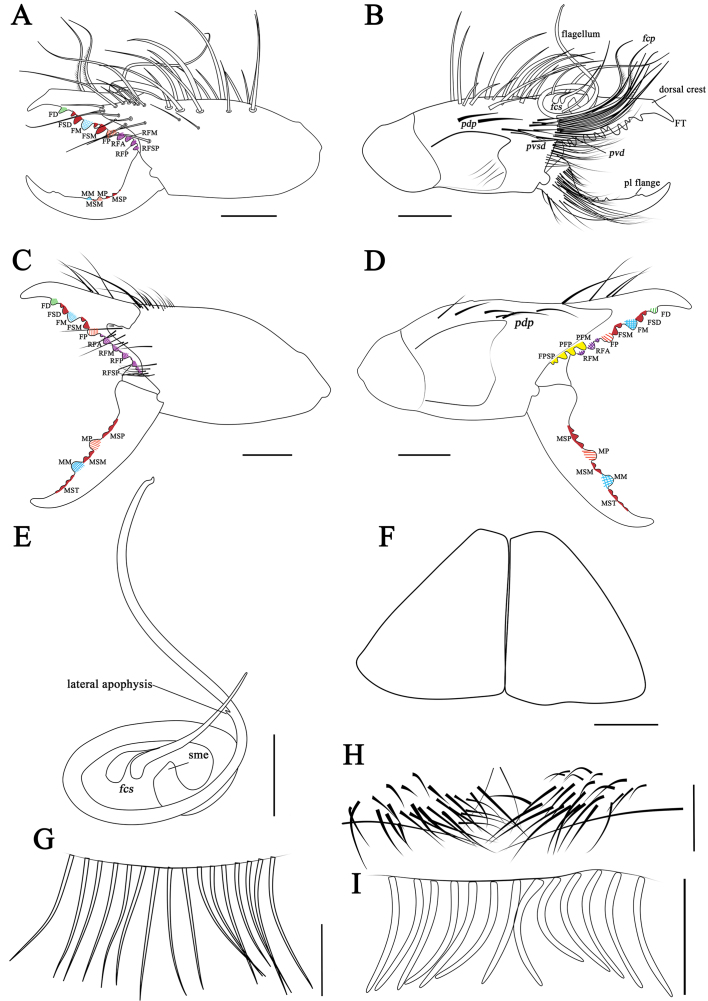
Karschia (Karschia) shannan sp. nov.: **A** Male left chelicera, retrolateral aspect; **B** Male left chelicera, prolateral aspect; **C** Female left chelicera, retrolateral aspect; **D** Female left chelicera, prolateral aspect; **E** Male flagellum and *fcs.* **F** Female genital operculum, ventral aspect; **G** Female ctenidia on sternite IV, ventral aspect; **H** Male ctenidia on sternite Ⅲ, ventral aspect; **I** Male ctenidia on sternite IV, ventral aspect. Scale bars = 1.0 mm (A–D); 0.5 mm (E–I).

Adult male (holotype) (Fig. 1A, B, Figs 2, 3, Fig. 10A, B and E).

**Measurements.** Total body length 19.16, CH 1.84, CL 5.67, CW 1.72, PL 2.67, PW 3.31, A/CP 6.84, CL/CH 3.08. Pedipalp 19.42 (4.21, 6.80, 4.28, 1.14), leg Ⅰ 14.45 (2.97, 4.17, 2.48, 1.20), leg Ⅱ 12.74 (2.81, 3.25, 1.98, 0.98), leg Ⅲ 16.06 (3.51 4.34, 2.35, 0.96), leg Ⅳ 23.06 (4.33, 7.04, 3.63, 1.72).

**Coloration.** General body colouration pale yellowish with opisthosoma slightly darker; a chestnut pigmentation may be present on distal areas of the propeltidium, on the pedipalps (from the femur to the tarsus) and dorsal surface of the legs (from the femur to the tarsus) (Fig. 1A and B). Cheliceral manus predominantly yellowish, with some black areas and, in retrolateral view, with three black longitudinal stripes; cheliceral fingers with apices burnt umber (Fig. 2A and B). Opisthosomal tergites black and the side of sternites black (Fig. 1A and B).

**Prosoma.** Propeltidium wider than long (i.e. ratio of PL/PH 0.81), with very thin, ﬁliform hair-like setae and some bifurcated tip setae of different sizes perpendicular to the surface of the propeltidium (Fig. 2D). Anterior arci with four spiniform setae of different sizes on each side of ocular tubercle; posterior arci bears a row of 12 longer, thin, ﬁliform setae (partial shedding) (Fig. 2D). Ocular tubercle with four anterior medial spiniform setae, one central medial spiniform seta and two posterior medial spiniform setae (Fig. 2D). Lateral lobes partially fused to the propeltidium and each lobe with one spiniform seta (Fig. 2D). Mesopeltidium and metapeltidium wider than long; mesopeltidium rhomboidal; metapeltidium rectangular.

**Cheliceral dentition and processes.** Fixed finger with median teeth series comprising three primary teeth (FP, FM and FD), graded as FD﹤FP﹤FM, plus two secondary FSM teeth and two secondary FSD teeth; profondal teeth series with four teeth (a very reduced PFM, a tiny PFP, two tiny PFSP) (not annotated on Figs.); retrofondal teeth series with four teeth (RFA, RFM, RFP, RFSP); mucron with a small dorsal crest and apex (FT tooth) curved and hook-shaped (Fig. 2A, B, Fig. 3A and B). Movable ﬁnger with median teeth series comprising two primary teeth (MM and MP), plus two secondary MSM and three MSP teeth; mucron with a centrally depressed prolateral flange (Fig. 2A, B, Fig. 3A and B).

**Cheliceral setose areas and stridulatory plate.** Retrolateral and dorsal surface of the manus with large bifurcated tip setae and short simple tip bristle-like setae (*rlm* series); retrolateral and dorsal surfaces of the ﬁxed ﬁnger with simple tip setae of different sizes (*rlf* series) (Fig. 2A and Fig. 3A); prolateral surface with an array of setal types, as follows: proventral distal setae (*pvd*) consisting of two rows of plumose setae, the ventral-most reaching the distal PFSP tooth and the dorsal-most reaching the prolateral interdigital condyle (*pic*); two *fcp* (*i.e.* modified *pvd*); proventral subdistal setae (*pvsd* comb) forming a single row with about 13–15 spindly setae; the promedial setae (*pm*) series made up of thin, barbed, simple tipped bristle-like setae; the proximal prodorsal area is covered with some long, simple tipped, non-barbed bristle-like setae (*pdp*); stridulatory apparatus with 4 well-developed ridges occupying the anterior ventral region of the stridulatory plate (Fig. 2B and Fig. 3B). Distal limit of the prolateral setose area of the movable finger reaching the MSM tooth; movable finger prodorsal (*mpd*) setal series consisting of plumose setae arranged in a rather staggered row, adjacent to abundant non-plumose setae of the movable finger promedial (*mpm*) and proventral (*mpv*) setal series (Fig. 2B and Fig. 3B).

**Cheliceral flagellar complex.** Of the composite type, sessile, without fringe, but with a very small lateral apophysis (Fig. 2C and Fig. 3E). Flagellum rotatable, long, rolled and structure immovably attached prodorsally to the fixed finger (Fig. 2B and Fig. 3B). Two medium-length flagellar complex plumose (*fcp*) setae located ventrally to the flagellum and two swollen-based flagellar complex spiniform (*fcs*) setae arising dorsoproximally to the flagellar attachment point (Fig. 2B, C, Fig. 3B and E).

**Opisthosoma.** Entire surface covered with almost adpressed fine setae and numerous long, curved, bifurcate setae and tergites with abundant setae. Sternite Ⅲ with two posterior paramedian groups of needle-like ctenidia, being gradually larger towards the posterior margin (Fig. 2E and Fig. 3H); sternite Ⅳ with a row of 13 short and thick columnar ctenidia (Fig. 2F and Fig. 3I).

**Pedipalps**. All segment covered with short fine setae and long, thick setae. Tarsus ventrally with nine short strong spines without symmetrical arrangement; metatarsus with seven ventral spines, 2/1/2/1/1 pattern and with densely packed papillae (Fig. 10A and B).

**Legs.** Totally covered with long, thick setae and short setae. Leg Ⅰ spineless with two small claws. Tibiae Ⅱ, Ⅲ and Ⅳ with a pair of distal spiniform setae ventrally, tibiae Ⅱ and Ⅲ each with one distal spiniform seta dorsally. Metatarsi Ⅱ and Ⅲ with a series of three dorsal spiniform setae, a pair of distal spiniform setae ventrally and some paired short, thick, spiniform setae over their entire ventral surface, metatarsus Ⅳ also with these paired spiniform setae over its entire ventral surface and two distal spiniform setae ventrally. Additionally, ventral coxae of leg Ⅲ with special tubular setae (Fig. 10E): which are absent in other congeners, instead with only fine and bifurcate setae (e.g. Fig. 10F).

**Figure 4.**
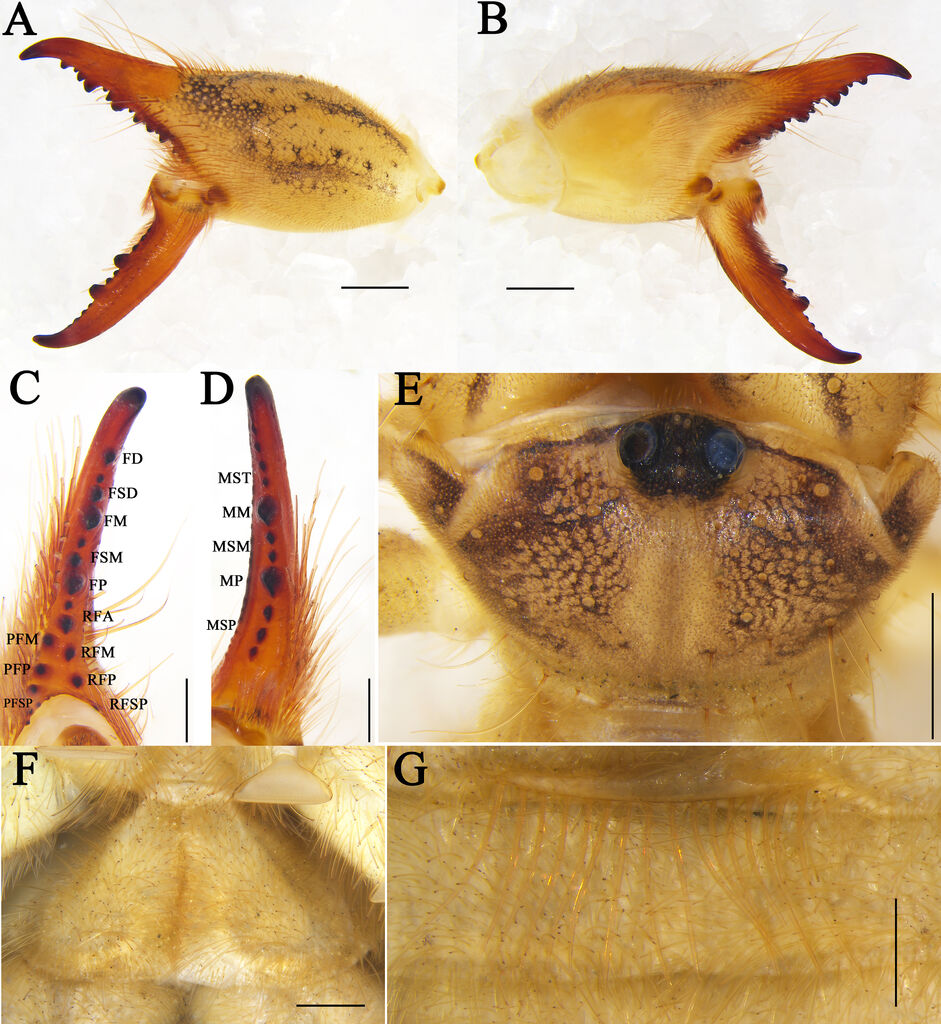
Karschia (Karschia) shannan sp. nov., female paratype: **A** Left chelicerae, retrolateral aspect; **B** Left chelicerae, prolateral aspect; **C** Dentition on left cheliceral fixed finger, ventral aspect; **D** Dentition on left cheliceral movable finger, ventral aspect; **E** Propeltidium, dorsal aspect; **F** Genital operculum, ventral aspect; **G** Ctenidia on sternite IV, ventral aspect. Scale bars = 1.0 mm (A, B, E); 0.5 mm (C, D, F, G).

**Adult female** (paratype female) (Fig. 1C, D, Figs 3, 4). Mostly same as males, except where noted.

**Measurements.** Total body length 18.02, CH 2.36, CL 6.51, CW 2.01, PL 2.65, PW 4.36, A/CP 5.28, CL/CH 2.76. Pedipalp 15.62 (3.01, 4.74, 3.64, 1.39), leg Ⅰ 11.03 (2.41, 3.42, 2.03, 0.89), leg Ⅱ 9.37 (1.60, 2.59, 1.09, 0.93), leg Ⅲ 12.94 (2.79, 3.12, 1.73, 1.13), leg Ⅳ 21.74 (4.56, 6.09, 3.65, 1.53).

**Coloration.** General body colouration as the male (Fig. 1C and D).

**Cheliceral dentition and processes.** Fixed finger with median teeth series comprising three well-developed primary teeth (FP, FM and FD), FD tooth smaller, while FM and FP similarly sized, plus two secondary FSD and two FSM teeth, all secondary teeth smaller than primary teeth, distal-most of each pair smallest; retrofondal teeth series uninterrupted with five teeth (RFSP, RFP, RFM, two RFA); profondal teeth series with five teeth (three PFSP, PFP, PFM) (Fig. 3C, D, Fig. 4A, B and C). Movable ﬁnger with median teeth series comprising two primary teeth (MM and MP), both similar in size, plus four secondary MST, two secondary MSM and three secondary MSP teeth (Fig. 3C, D, Fig. 4A, B and D).

**Opisthosoma.** Genital operculum typical triangular-shaped and with no clear demarcation between the plates; the rear edge of the genital sternite not chitinised and without clear opening (Fig. 3F and Fig. 4F). Sternite Ⅲ without ctenidia; only sternite Ⅳ with a row of 16 long and needle-like ctenidia, extending across the entirety of the adjacent sternite, reaching its posteror border. (Fig. 3G and Fig. 4G).

**Pedipalps**. Tarsus and metatarsus without spines and papillae.

**Legs.** Coxae of leg Ⅲ without special tubular setae.

#### Diagnosis

*Karschiashannan* sp. nov. differs in the male from all *Karschia* species by the ventral coxae of leg Ⅲ with special tubular setae (Fig. 10E). The new species can be further diagnosed by their pedipalpal metatarsi with densely packed papillae (Fig. 10B), the apex of their cheliceral fixed finger (FT) tapering (Fig. 2B and Fig. 3B), the reduced number of ctenidia on sternite Ⅳ - only with 13 short and thick columnar ctenidia (Fig. 2F and Fig. 3I) and flagellum proximally with a small lateral apophysis; flagellar complex plumose setae (*fcp*) medium-length (Fig. 2B, C, Fig. 3B and E). Females can diagnosed by the genital operculum, which, although typically triangular-shaped, has no clear demarcation between the genital plates, while the genital opening is not visible between, nor distal to the genital plates (Fig. 4F and Fig. 3F). Otherwise, females have long (i.e. reaching the posterior border of the adjacent sternite) and needle-like ctenidia on sternite Ⅳ (Fig. 4G and Fig. 3G).

#### Etymology

Noun in apposition, taken from Shannan City where this type materials of the new species were collected.

#### Distribution

**Figure 5.**
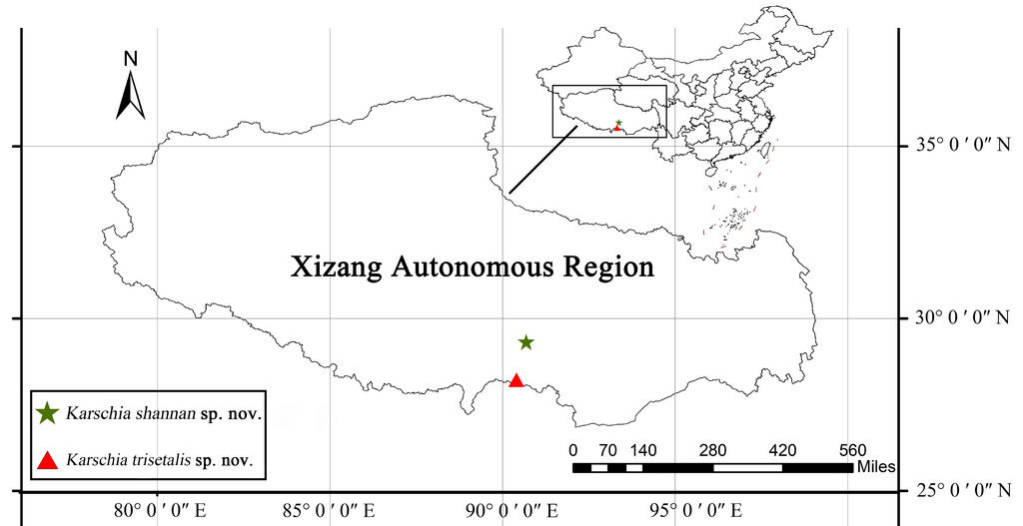
Map plotting known locality records.

China (Xizang) (Fig. 5).

### Karschia (Karschia) trisetalis sp. nov.

C4EBAA4F-2611-532F-B451-2DBA228DD8BA

8626AC24-D496-4359-9A0F-238D89209C29

#### Materials

- **Type status:** Holotype. **Occurrence:** recordedBy: Zhiyong Di; sex: male; lifeStage: adult; occurrenceID: 084080C6-C1DB-5626-A46C-C7C5452E9050; **Taxon:** scientificName: Karschia (Karschia) trisetalis; **Location:** country: China; stateProvince: Xizang Autonomous Region; county: Luozha; municipality: Shannan; locality: Zhari Town; verbatimElevation: 4200 m; verbatimCoordinates: 28.2217°N, 90.4011°E; **Identification:** identifiedBy: Wenlong Fan; **Event:** year: 2021; month: August; day: 6; **Record Level:** institutionID: the Museum of Hebei University; institutionCode: MHBU-Sol-XZ2021080601
- **Type status:** Paratype. **Occurrence:** recordedBy: Zhiyong Di; sex: 1 male, 1 female; lifeStage: adult; occurrenceID: FE77A507-E754-5057-8914-A5E32447E218; **Taxon:** scientificName: Karschia (Karschia) trisetalis; **Location:** country: China; stateProvince: Xizang Autonomous Region; county: Luozha; municipality: Shannan; locality: Zhari Town; verbatimElevation: 4200 m; verbatimCoordinates: 28.2217°N, 90.4011°E; **Identification:** identifiedBy: Wenlong Fan; **Event:** year: 2021; month: August; day: 6; **Record Level:** institutionID: the Museum of Hebei University; institutionCode: 1 male MHBU-Sol-XZ2021080602 and 1 female MHBU-Sol-XZ2021080603

#### Description

**Figure 6.**
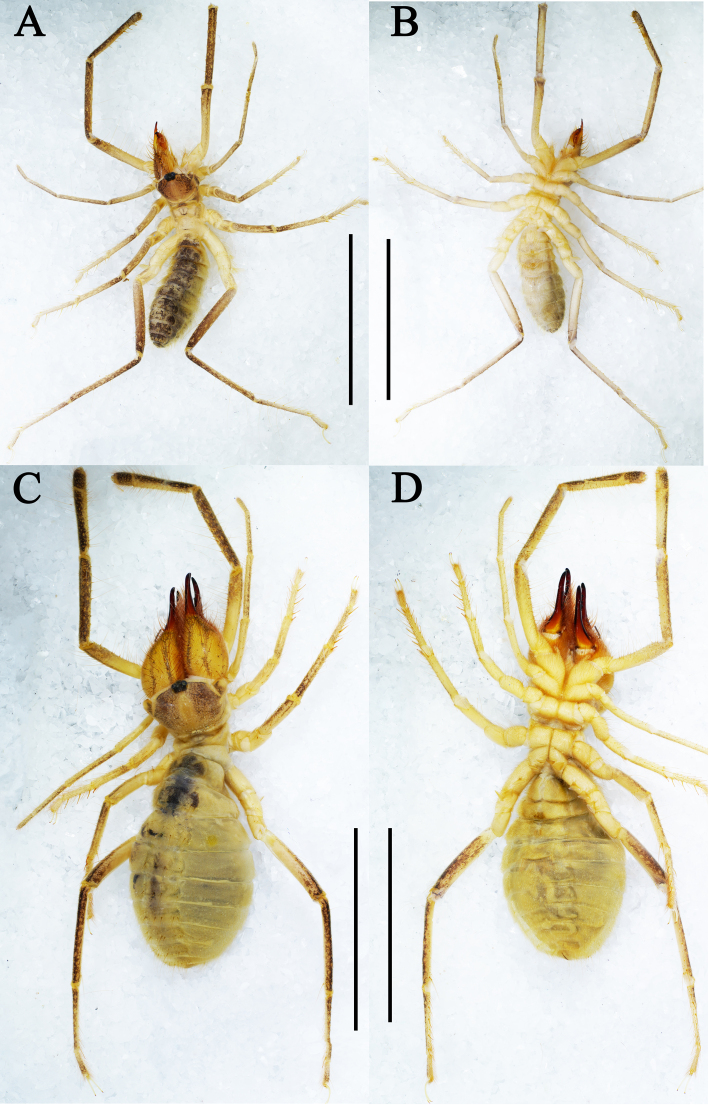
Habitus. Karschia (Karschia) trisetalis sp. nov., habitus: **A, B** Male holotype; **C, D** Female paratype. Scale bars: 8 mm.

**Figure 7.**
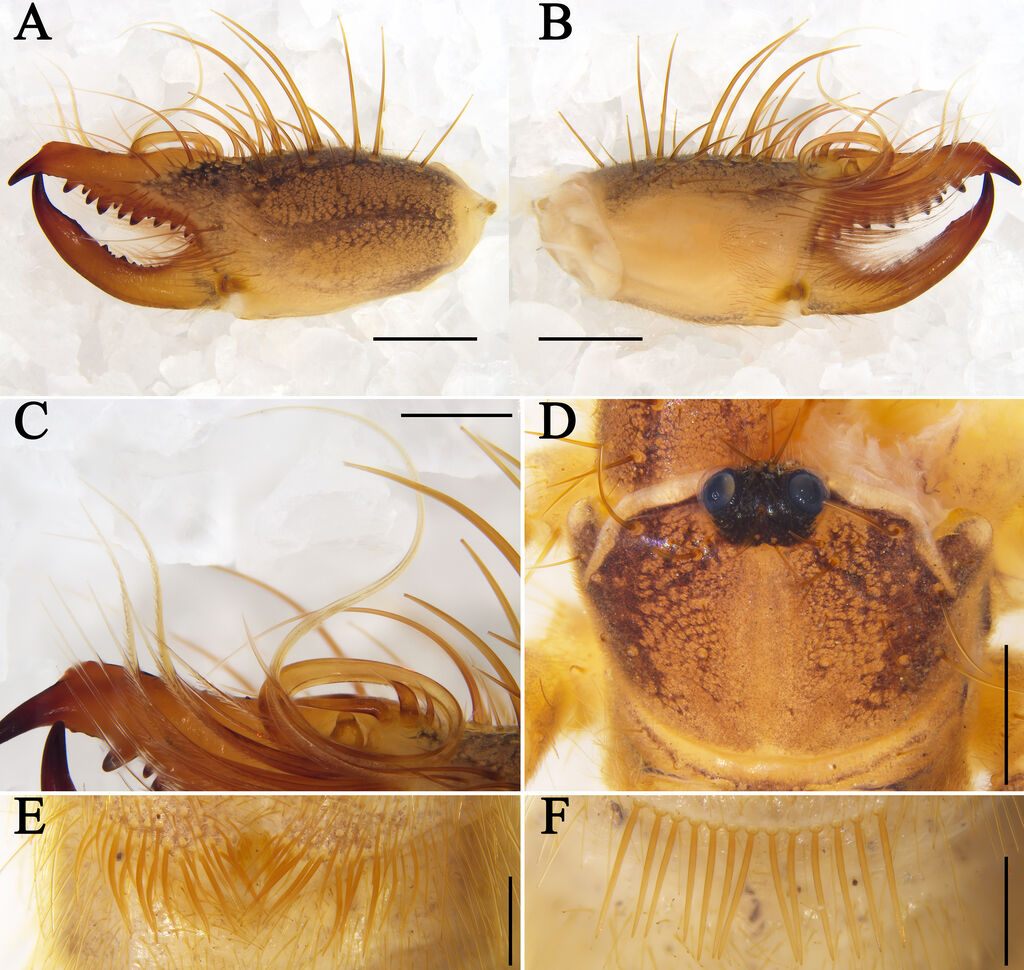
Karschia (Karschia) trisetalis sp. nov., male holotype. **A** Left chelicerae, retrolateral aspect; **B** Left chelicerae, prolateral aspect; **C** Flagellar complex, prolateral aspect; **D** Propeltidium, dorsal aspect. **E.** Ctenidia on sternite Ⅲ, ventral aspect; **F** Ctenidia on sternite IV, ventral aspect. Scale bars = 1.0 mm (A, B, D); 0.5 mm (C, E, F).

**Figure 8.**
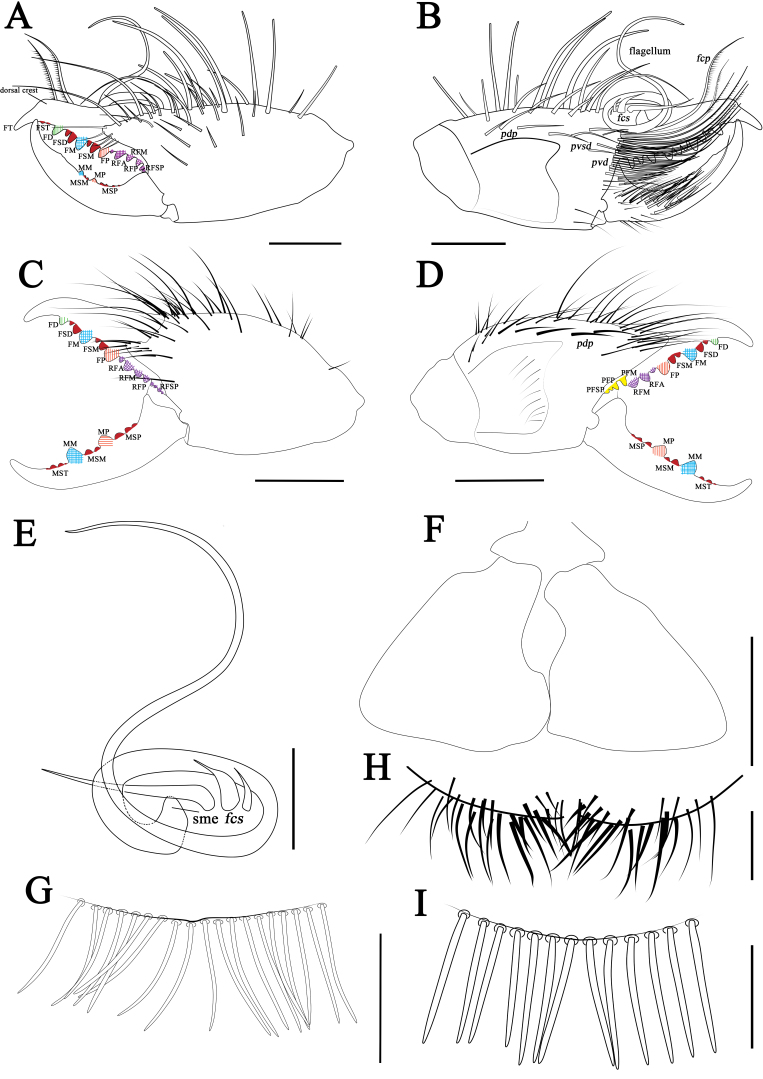
Karschia (Karschia) trisetalis sp. nov.: **A** Male left chelicera, retrolateral aspect; **B** Male Left chelicera, prolateral aspect; **C** Female left chelicera, retrolateral aspect; **D** Female left chelicera, prolateral aspect; **E** Male flagellum and *fcs*; **F** Female genital operculum, ventral aspect; **G** Female ctenidia on sternite IV, ventral aspect; **H** Male ctenidia on sternite Ⅲ, ventral aspect; **I** Male ctenidia on sternite IV, ventral aspect. Scale bars = 0.5 mm (E, H-I); 1.0 mm (A, B, F); 2.0 mm (C, D).

Adult male (holotype) (Fig. 6A, B, Figs 7, 8, Fig. 10C, D and F).

**Measurements.** Total body length 16.53, CH 1.65, CL 4.67, CW 1.49, PL 2.09, PW 3.04, A/CP 8.16, CL/CH 2.83. Pedipalp 18.68 (4.26, 5.56, 3.99, 2.1), leg Ⅰ 13.57 (2.95, 3.69, 2.37, 1.19), leg Ⅱ 10.79 (2.09, 2.82, 1.97, 0.92), leg Ⅲ 14.76 (3.02, 3.78, 2.86, 0.98), leg Ⅳ 22.92 (5.03, 5.82, 4.22, 1.51).

**Coloration.** General body colouration pale yellowish with opisthosoma slightly darker; a chestnut pigmentation may be present on distal areas of the propeltidium, on the pedipalps (from the femur to the tarsus) and dorsal surface of the legs (from the femur to the tarsus) (Fig. 6A and B). Cheliceral manus predominantly yellowish, with some black areas and, in retrolateral view, with three black longitudinal stripes; cheliceral fingers russet with apices burnt umber (Fig. 7A and B). Opisthosomal tergites black and the side of sternites black.

**Prosoma.** Propeltidium wider than long ratio of (i.e. PL/PH 0.69), with very thin, ﬁliform hair-like setae and some bifurcated tip setae of different sizes perpendicular to the surface of the propeltidium (Fig. 7D). Anterior arci with four spiniform setae of different sizes on each side of ocular tubercle, posterior arci bears a row of ten longer, thin, ﬁliform setae. Ocular tubercle with four anterior medial spiniform setae, one central medial spiniform seta and two posterior medial spiniform setae (Fig. 7D). Lateral lobes partially fused to the propeltidium and each lobe with one spiniform seta. Mesopeltidium and metapeltidium wider than long; mesopeltidium rhomboidal; metapeltidium rectangular.

**Cheliceral dentition and processes.** Fixed finger with median teeth series comprising three primary teeth (FP, FM and FD), graded as FD﹤FP﹤FM; plus two secondary FSM, two secondary FSD and notably two tiny secondary FST teeth; profondal teeth series with four teeth (one tiny PFM, one tiny PFP, two tiny PFSP); retrofondal teeth series with four teeth (RFA, RFM, RFP, RFSP); mucron with a larger dorsal crest and apex (FT tooth) tapering, curved and hook-shaped (Fig. 7A, B, Fig. 8A and B). Movable ﬁnger with median teeth series comprising two primary teeth (MM and MP), two secondary MSM and four secondary MSP teeth; mucron without distinct prolateral flange (Fig. 7A, B, Fig. 8A and B).

**Cheliceral setose areas and stridulatory plate.** Retrolateral and dorsal surface of the manus with large bifurcated tip setae and short simple tip bristle-like setae (*rlm* series); retrolateral and dorsal surfaces of the ﬁxed ﬁnger with simple tip setae of different sizes (*rlf* series) (Fig. 7A and Fig. 8A); prolateral surface with an array of setal types as follows: proventral distal setae (*pvd*) consisting of two rows of plumose setae, the ventral-most reaching the distal PFSP tooth and the dorsal-most reaching the prolateral interdigital condyle (*pic*); two *pvd* modified (= *fcp*); proventral subdistal setae (*pvsd* comb) forming a single row of only a few (i.e. 4–6) spindly setae; the promedial setae (*pm*) series made up of thin, barbed, simple tip bristle-like setae of reduced numbers; the proximal prodorsal area is covered with some long, simply tipped, non-barbed bristle-like setae (*pdp*); stridulatory apparatus without well-developed ridges, a clear dent occupying the ventral region of the stridulatory plate (Fig. 7B and Fig. 8B). Distal limit of the prolateral setose area of movable finger reaching the level of MSM tooth; movable finger prodorsal (*mpd*) setal series consisting of plumose setae arranged in a rather staggered row, adjacent to abundant non-plumose setae of the movable finger promedial (*mpm*) and proventral (*mpv*) setal series (Fig. 7B and Fig. 8B).

**Cheliceral flagellar complex.** Of the composite type, sessile and without fringe and lateral apophysis (Fig. 7C and Fig. 8E). Flagellum rotatable, long, rolled and structure immovably attached prodorsally to the fixed finger (Fig. 7C and Fig. 8E). Two medium-length flagellar complex plumose (*fcp*) setae located ventrally to the flagellum and three basally-swollen flagellar complex spiniform (*fcs*) setae arising dorsoproximally to the flagellar attachment point, the anterior-most angled forward in line with the fixed finger (Fig. 7B, C and Fig. 8E).

**Opisthosoma.** Entire surface covered with almost adpressed fine setae and numerous long, curved, bifurcate setae and tergites with abundant setae. Sternite Ⅲ with two posterior paramedian groups of long needle-like ctenidia, being gradually larger to posterior (Fig. 7E and Fig. 8H); sternite Ⅳ with a row of 13 short and thick columnar ctenidia (Fig. 7F and Fig. 8I).

**Pedipalps**. All segments covered with short fine setae and long, thick setae. Tarsus ventrally with eight short and strong spines, not symmetrical arrangement; metatarsus with four ventral spines above the papillae, not symmetrical arrangement and with thin sparse papillae (Fig. 10D).

**Legs.** Leg I coated with abundant short setae similar to those on pedipalps, without stout or spiniform setae; tibia and metatarsus with few long thin setae, tarsus without spiniform setae; with two small claws. Tibiae Ⅱ and Ⅲ each with one distal spiniform seta dorsally and a pair of distal spiniform setae ventrally. Metatarsi Ⅱ and Ⅲ with a series of three spiniform setae and a pair of distal spiniform setae ventrally; metatarsus Ⅳ ventrally with two distal spiniform setae and three to five thick, spiniform setae arranged in a prolateral row. Legs totally covered with long, thick setae and short fine setae. Distal part of claw short, occupying approximately 1/5 of the claw length.

**Figure 9.**
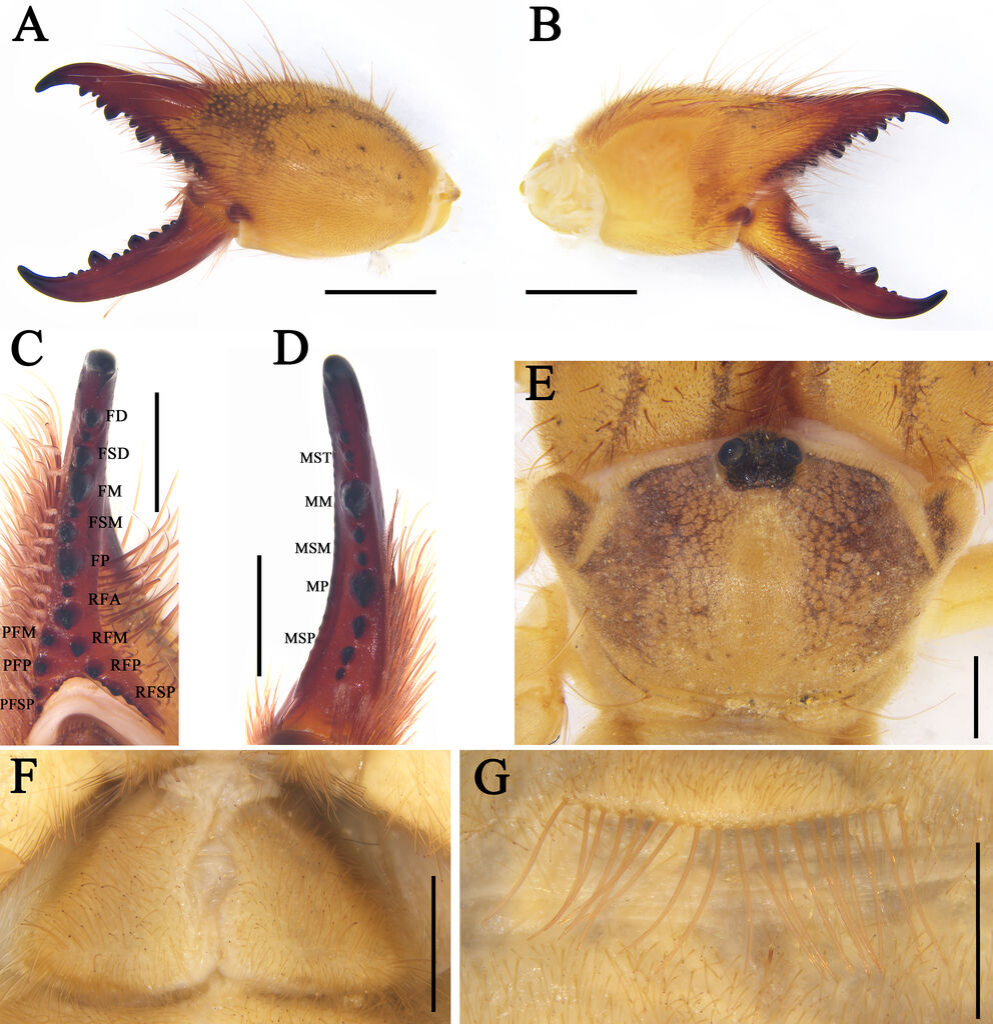
Karschia (Karschia) trisetalis sp. nov., female paratype: **A** Left chelicerae, retrolateral aspect; **B** Left chelicerae, prolateral aspect; **C** Dentition on left cheliceral fixed finger, ventral aspect; **D** Dentition on left cheliceral movable finger, ventral aspect; **E** Propeltidium, dorsal aspect; **F** Genital operculum, ventral aspect. **G** Ctenidia on sternite IV, ventral aspect. Scale bars = 0.5 mm (C, G); 1.0 mm (E, F); 2.0 mm (A, B).

**Figure 10.**
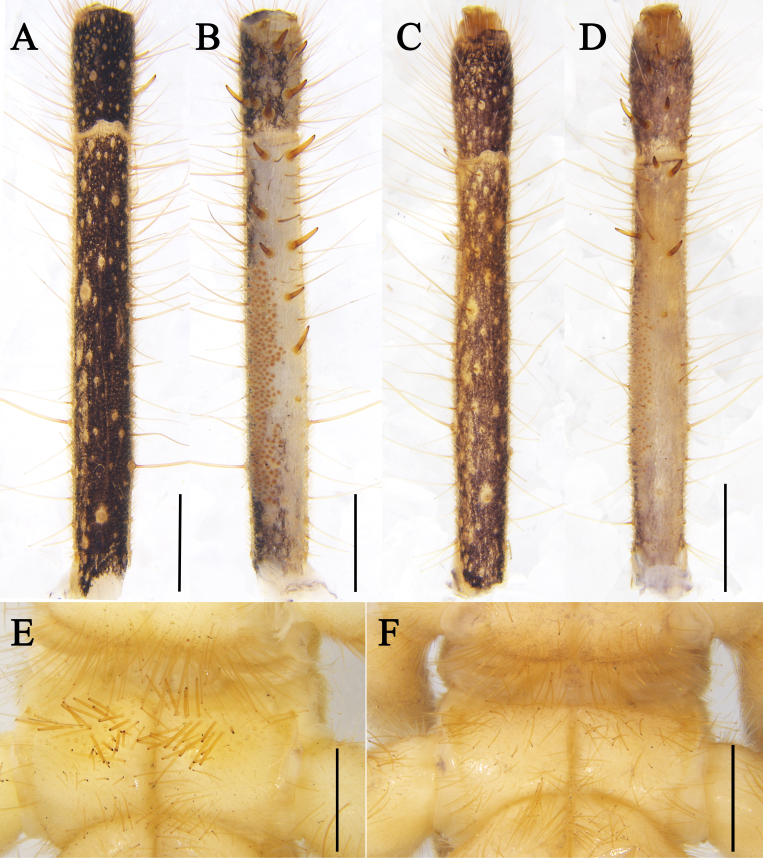
**A, B, E.** Karschia (Karschia) shannan **sp. nov.**, holotype male. **C, D, F.** Karschia (Karschia) trisetalis **sp. nov.**, holotype male. **A–D**. Left pedipalpal metatarsus and tarsus. **E, F.** coxa of leg Ⅲ.

**Adult female** (paratype female) (Fig. 6C, D and Figs 8, 9). Mostly same as males, except where noted.

**Measurements.** Total body length 25.53, CH 3.12, CL 7.69, CW 2.67, PL 3.31, PW 5.45, A/CP 4.88, CL/CH 2.46. Pedipalp 18.84 (4.84, 5.60, 4.07, 1.56), leg Ⅰ 13.15 (2.35, 3.75, 2.40, 1.12), leg Ⅱ 13.06 (1.82, 2.75, 1.78, 2.75), leg Ⅲ 14.21 (2.93, 3.17, 2.15, 1.43), leg Ⅳ 21.70 (4.27, 5.70, 3.27, 1.57).

**Coloration.** General body colouration as the male (Fig. 6C and D), except cheliceral manus in retrolateral view only with one black longitudinal stripe (Fig. 9A).

**Cheliceral dentition and processes.** Fixed finger with median teeth series comprising three well-developed primary teeth (FP, FM and FD), FD tooth size smaller, while FM and FP of similar size, plus two secondary FSD and two secondary FSM, all smaller than primary teeth, distal-most of each pair smallest, while the largest of each pair approaches the size of FD; retrofondal teeth series uninterrupted with nine teeth (five RFSP, RFP, RFM, two RFA); profondal teeth series consisting of four teeth (two PFSP, PFP, PFM) (Fig. 8C, D, Fig. 9A, B and C). Movable ﬁnger with median teeth series comprising two primary teeth (MM and MP), similar in size, plus three secondary MST, two secondary MSM and three MSP teeth (Fig. 9A, B and D).

**Chelicera stridulatory plate.** Stridulatory apparatus anteriorly with well-developed ridges (Fig. 8D and Fig. 9B).

**Opisthosoma.** Genital operculum typical triangular-shaped with clear demarcation between the plates; the central region being deeply indented on each side, whilst the posteror edge of the genital sternite is not chitinised and without clear opening (Fig. 8F and Fig. 9F). Sternite Ⅲ without ctenidia; only sternite Ⅳ with a row of 19 moderately-long and needle-like ctenidia centrally (Fig. 8G and Fig. 9G).

**Pedipalps**. Tarsus and metatarsus without spines and papillae.

#### Diagnosis

*Karschiatrisetalis* sp. nov. differs in the male from other all *Karschia* species by the flagellar complex with three flagellar complex spiniform setae (*fcs*) (Fig. 7C and Fig. 8E). Males of *Karschiatrisetalis* sp. nov. can also be diagnosed by the cheliceral fixed finger with two tiny subterminal teeth (FST) (Fig. 7A, C and Fig. 8A). Females can be easily differentiated from other *Karschia* species by the central region of the genital plates deeply indented and genital opening visible between plates (Fig. 8F and Fig. 9F).

#### Etymology

The specific name is an adjectival combination of the Latin words “*tri*” (prefix from *trēs*, meaning three) with the noun “*seta*” (i.e. spiniform structures), plus the suffix "-*ālis*" (neuter -*āle*), Together, the name means "pertaining to three setae" as an adjective, referring to the flagellar complex of male chelicerae with three *fcs*.

#### Distribution

China (Xizang) (Fig. 5).

## Supplementary Material

XML Treatment for Karschia (Karschia) shannan

XML Treatment for Karschia (Karschia) trisetalis
